# Assessment of factors associated with complete immunization coverage in children aged 12-23 months: a cross-sectional study in Nouna district, Burkina Faso

**DOI:** 10.1186/1472-698X-9-S1-S10

**Published:** 2009-10-14

**Authors:** Aboubakary Sanou, Seraphin Simboro, Bocar Kouyaté, Marylène Dugas, Janice Graham, Gilles Bibeau

**Affiliations:** 1 Université de Montréal, Department of Social and Preventive Medicine, Québec, Canada; 2 Centre de Recherche en Santé de Nouna, Burkina Faso; 3 Centre National de Recherche et de Formation sur le Paludisme, Ouagadougou, Burkina Faso; 4 Dalhousie University, Bioethics, Faculty of Medicine, Halifax, Canada; 5 Department of Anthropology, Université de Montréal, Québec, Canada

## Abstract

**Background:**

The Expanded Program on Immunization (EPI) is still in need of improvement. In Burkina Faso in 2003, for example, the Nouna health district had an immunization coverage rate of 31.5%, compared to the national rate of 52%. This study identifies specific factors associated with immunization status in Nouna health district in order to advance improved intervention strategies in this district and in those with similar environmental and social contexts.

**Methods:**

A cross-sectional study was undertaken in 41 rural communities and one semi-urban area (*urban in the text*). Data on 476 children aged 12 to 23 months were analyzed from a representative sample of 489, drawn from the Nouna Health Research Centre's Demographic Surveillance System (DSS) database. The vaccination history of these children was examined. The relationships between their immunization status and social, economic and various contextual variables associated with their parents and households were assessed using Chi square test, Pearson correlation and logistic regression.

**Results:**

The total immunization coverage was 50.2% (CI, 45.71; 54.69). Parental knowledge of the preventive value of immunization was positively related to complete immunization status (p = 0.03) in rural areas. Children of parents who reported a perception of communication problems surrounding immunization had a lower immunization coverage rate (p < 0.001). No distance related difference exists in terms of complete immunization coverage within villages and between villages outside the site of the health centres. Children of non-educated fathers in rural areas have higher rates of complete immunization coverage than those in the urban area (p = 0.028). Good communication about immunization and the importance of availability of immunization booklets, as well as economic and religious factors appear to positively affect children's immunization status.

**Conclusion:**

Vaccination sites in remote areas are intended to provide a greater opportunity for children to access vaccination services. These efforts, however, are often hampered by the poor economic conditions of households and insufficient communication and knowledge regarding immunization issues. While comprehensive communication may improve understanding about immunization, it is necessary that local interventions also take into account religious specificities and critical economic periods. Particular approaches that take into consideration these distinctions need to be applied in both rural and urban settings.

**Abstract in French:**

See the full article online for a translation of this abstract in French.

## Abstract in French

See Additional file 1 for a translation of the abstract to this article in French.

## Background

Immunization has a long history of success. Studies have shown that it has an impact on the major causes of infant death and that it shapes trends of mortality and morbidity among communities [1,2]. Immunization remains one of most cost-effective health interventions [3,4] and has proven to prevent up to 24% of the 10 million yearly deaths of children under five [5]. Nevertheless, vaccination has always faced multiple adversities [6-11], the most recent being the suspicion that it is an international conspiracy against selected communities, particularly those in developing countries [12-15].

To benefit from its full potential, including the positive externalities for non-immunized children, the World Health Organization (WHO) suggests that complete vaccination coverage should reach at least 90% of children at the country level and 80% in sub-areas by the year 2010 [16]. Such an ambitious objective is far beyond the actual reach of most developing countries for several reasons. While in the Netherlands, for example, the perception of risk of infection is a determining factor in the decision of Dutch parents to vaccinate their children [17], daily living conditions determine whether parents seek immunization for their children in many developing regions. In Kinshasa, for example, there is a reported strong association between specific vaccines and mothers' education level [18]. Higher socio-economic condition of the parents is also associated with greater probability of the child being vaccinated under a routine vaccination program compared to mass vaccination campaigns [19]. Knowing what is at stake is also important in vaccination seeking behaviour [20]; not participating in immunization sessions appears to be linked to lack of information amongst parents [21] or to a deliberate choice to refuse [17,22]. Residing near health facilities has also been considered a strong determinant for getting good vaccination coverage [23,24]. In reality, however, this is not always the case since areas in the vicinity of health services often show weak immunization coverage [25].

Ethnic disparities in vaccination rates have been reported in countries like Mali, Niger and Senegal, where, respectively, the Bambaras, Djermas-Songhais and Sérères appear to have more complete immunization coverage [26]. Similarly, in the United States, racial and ethnic differences are reflected in influenza vaccine coverage [27]. Vaccination uptake is thus considered to be highly culturally sensitive, influenced by local perceptions on childhood diseases and decisional processes in households [28]. While some authors emphasize that vaccination uptake has a cultural foundation in some African communities[11,29-33], others attribute low achievement of immunization to cultural discrepancies [6,34-36] and some etiological considerations regarding preventable diseases have even been identified as "cultural prejudice" [12].

While the literature shows the importance of social, economic, geographic and cultural factors in the vaccination status of a child, achieving adequate vaccination coverage is not only related to the attitudes and capabilities of parents. Researchers have demonstrated that the organization and functioning of the health care system and services, including the ways health workers perform their activities, constitute key elements in vaccination coverage: it is known that the manner in which immunization activities are organized and services are delivered [6], and the interaction between parents and health workers [9,31] greatly influence the immunization coverage. Unfortunately these aspects are not always taken into consideration by health workers or by the planners of vaccination services.

The quality of the service at the health post with regards to reception, waiting time [24,31], and good clinical practice [37] also affect demand for immunization.

The success of immunization activities is also associated with the strategies used to reach target populations and to deliver service. Generally, two major health service strategies are utilized complementarily: i) routine vaccination activities are performed by using a combination of mobile and fixed-point strategies or advanced strategies for remote villages; and ii) targeted campaigns are undertaken to complement routine activities and to avoid the emergence of specific epidemics (particularly meningitis and measles). These latter campaigns are however said to be costly [38]. Immunization improvement can adopt *risk based strategies *(who should be vaccinated?) or *place based strategies *(where to vaccinate?) [5]. Strategies that enhance immunization coverage also include approaches that improve demand for immunization, address access to immunization services, compulsory immunization, and adopt provider based strategies [39]. Any combination of these approaches is also possible [40,41].

The Expanded Program on Immunization (EPI) is far from achieving the success experienced by the smallpox eradication program, which is said to have inspired the launching of the EPI in 1974 [42]. In Burkina Faso in 2003, the complete immunization coverage of children aged 12 to 23 months for the six preventable diseases targeted by the EPI was 52%; 3% of these children had never been vaccinated. Sub-area coverage varies greatly, ranging from 18% to 79%. The Nouna health district, for its part, had one of the lowest complete immunization coverage rates, 31.5%, and 5.2% of children aged 12 to 23 months had received no vaccinations. [22].

Through an operational research grant provided by the International Development Research Centre (IDRC) as part of the Canadian International Immunization Initiative Phase 2 (CIII2), an intervention was planned by the Centre de Recherche en Santé de Nouna (CRSN) and the Nouna health district to address this low coverage rate. As part of the planning for that intervention, the CRSN carried out studies to assess the various social, cultural, anthropological and economic factors leading to non-vaccination as well as assess the actual vaccination coverage rates.

This article provides evidence of the issues that appear to be related to complete and incomplete immunization coverage when taking into account details of the communities and the existing structure and provision of health services. As can be seen from the diverse range of strategies reported above, improving immunization coverage in the Nouna district will require concrete knowledge and responsiveness to the particular issues associated with low coverage in this region. The results of the study reported here provide additional information for constructing local interventions to tackle problems of low coverage in the Nouna district as well as other areas with similar conditions. They will also be important for the 2006-2015 decade target of international institutions such as WHO and UNICEF to reach all disadvantaged areas [43].

### Study location

The study area is the health district of Nouna, in the North-West of Burkina Faso, about 300 km from Ouagadougou, the capital city. The district has a district hospital, the Centre de Santé avec Antenne chirurgicale (CMA) and 24 peripheral health centres called Centre de Santé et de Promotion Sociale (CSPS), with each of these peripheral health centres being run by a team of two to four health workers and one to three unqualified volunteers. The district hosts the CRSN, which includes a Demographic Surveillance System (DSS), and covers a population of around 70,000 in 57 villages and the town of Nouna with its seven sectors. The population is composed of several ethnic groups including two native groups (Marka and Bwaba) as well as the Samo, Mossi and Fulani. The people in the region are predominantly Muslim, with some percentage of the population being Christian [44,45]. Three quarters of the population is illiterate, dependent on subsistence farming and livestock breeding. The average family size is 10 individuals with some compounds composed of multiple generations. The principal language spoken is Djoula, used by almost all the ethnic groups. The study area includes the district hospital, the urban health centre and nine peripheral health centres. Epidemic and endemic diseases, some of which are preventable through vaccination, dominate the epi-demiological profile. Local cultural belief systems influence etiological explanations of diseases and health-seeking behaviour is dominated by traditional medicines. It should be noted that, in this area, malaria remains the primary cause of morbidity and mortality, particularly among children.

## Methods

### Sample and procedure

This research is a cross-sectional study planned as a pre-intervention assessment. A sample of 489 children aged 12 to 23 months was calculated using the Epi-Info Statcalc with a 95% confidence level, a power of 80%, and 46% as the estimated immunization coverage rate in the research area (this rate was estimated by the research team, based on their knowledge of the local context). The sampling was carried out using the database of the DSS of CRSN, which contained 2,508 households with children in the targeted age group. The number of households to visit in each village was determined according to the proportion of 12 to 23 month-old children in the village from the database. The codes of all the households of a village were written on pieces of paper and then the households were drawn (without replacement) until the required number for the village was obtained. The household was identified using this code and the name of the head of the household. Children were identified using their name and the name of the parents. An appointment was set with the parents; only one child was selected per household. Of 489 children selected, 13 children were not included in the analysis: two children had deceased, five households migrated with their children and the data quality checking procedures rejected four entries for insufficient information and two other children were excluded because they did not belong to the eligible age group. The analyses were performed with a final sample of 476 individuals. There were no refusals.

### The questionnaire and data collection

The questionnaire was built following an adapted household survey questionnaire that is used in the DSS; in addition, a number of focus group discussions were held a month before we framed the questionnaire. The revised questionnaire contains six categories of data: 1) identification of the household and the child; 2) family information extracted from the DSS data base; 3) socioeconomic status information; 4) perception about risk and decision of prevention; 5) birth place of the child and exposure to vaccination information; and 6) knowledge about immunization and participation in prevention sessions. The economic status of households was determined from information gained from interviewees. A basic or *core economical revenue *of the household was estimated on the basis of the revenue from the principal activity of the head of the household in addition to the assets (agricultural production, cattle and poultry) of the household. While we recognize that the *core economic resource *does not provide an account of the total financial resources of the household, it does provide a measurable economic indicator for comparison.

In a section dealing with the mother's participation in immunization activities - i.e. presenting at vaccination sites (vaccination sites are selected fixed places in villages where immunization take place) - and knowledge about immunization, information was also collected on immunization uptake (the principal dependant variable). Information was collected from immunization documents and from the mothers' statements, as suggested by many authors [46,47]. To guarantee the accuracy of this information, we examined multiple written sources used for immunization documentation including the immunization record cards, the prenatal consultation booklet of the mother, the infant files of the health centre and the immunization record book of the village health worker. Verbal information from the mother concerning each vaccine uptake was sought and a direct observation of the BCG scar was performed on the child. The final information obtained by the mother from health workers was also recorded. Responses such as: "*we've been told that our child was too old for this vaccine" *or *"the nurses said the child has got all his vaccines" *were used to correct the final immunization status. It should be noted that mothers give great importance to this final information.

The vaccination sessions are held in locations known as vaccination sites, identified by health workers and communities in the villages. The geographical coordinates of these vaccination sites were taken using a Global Positioning System (GPS). The distance between the household and the vaccination site was then ascertained for inclusion as a variable in the final analyses. The Average Theoretical Range of Action (ATRA) for health centres in 2004 in Burkina Faso was 8.3 km. This indicator was used for scaling the distance from the child's village to the health centre (0 = 0 km, representing residence in a village that has a health centre, 1 = residential distance between 1 km to 8.3 km away from the closest health centre, 2 = 8.4 to 16.6 km, 3 = 16.7 to 24.9 km and 4 ≥25 km). This was used to analyse the relationship between immunization uptake and residential distance from a health centre.

Taking into account the multiethnic characteristics of the study population, 18 representative interviewers participated in the data collection after a week-long training session. Two Masters level (MSc.) research coordinators supervised the interviewers. Both men and women were interviewed in the households. The completeness, logical structure and acceptability of the responses were checked in the field and at the office before we transferred the questionnaires for data entry.

### Data analysis

Data entry was performed using ACCESS and tables were transferred into Epi-Info and SPSS. Relative frequencies and other descriptive statistics were performed to present the distribution of the independent variables and vaccine uptake. Non-parametric analysis using *chi square test *was used to analyze the relation between the vaccination status of the child and the independent variables. A child was considered completely vaccinated if s/he received the BCG vaccine, the four doses of oral polio, three doses of DTP, measles and yellow fever vaccine. This study did not consider the validity of the dose of vaccines received (i.e. whether vaccines were administered in compliance with the vaccination schedule). After dichotomization, the independent variables were examined in a correlation analysis using Pearson's correlation coefficients to detect potential collinearities in the logistic regression and lead to appropriate data analysis and reporting [48,49]. The independent variables that showed significance for complete vaccination uptake were included in the logistic regression analyses. Given that in the rural areas many more variables proved significant in relation to the dependant variables, we excluded Nouna town from the regression analysis. Finally, interpretation of our findings takes into account the literature, context and purpose of the study.

## Results

### Characteristics of study participants

Table 1 indicates that the data relate to 476 children, 228 (47.9%) boys and 248 (52.1%) girls between 12 and 23 months of age. Children residing in the town of Nouna represented 26.7% (127) while the remaining came from surrounding villages. Nearly 40% of the children were born in health facilities and 38% were residing in a village hosting a health centre. The majority of fathers (53.4%) and mothers (74.6%) attended no school or had received Islamic religious teachings through Koranic schooling (respectively 14.9% and 8.6%). Some mothers attended adult literacy classes (10.7%). The majority of parents were Muslim (295/476), while Christians represented about 30% and local belief systems 8.6%. The dominant marital status was monogamous (56.1%) followed by polygamous (38.7%). Three ethnic groups composed the majority of the study population: Marka 43.1%, Bwaba 22.5% and Mossi 19.7%. The principal activity of the parents was agriculture (86.6%). The mean estimated core annual revenue of the households was 509373 CFA francs, ranging from 1500 CFA francs to 6600000 CFA francs (1 USD is worth approximately 450 CFA francs).

**Table 1:** Characteristics of study participants.

**Variables**	**Number of children (n)**	**Percentages (%)**
Sex of child		
Male	228	47.9
Female	248	52.1
Total	476	100.0
		
Availability of a vaccination record document		
No	127	26.7
Yes	349	73.3
Total	476	100.0
		
Locality		
Urban	127	26.7
Rural	349	73.3
Total	476	100.0
		
Birth place		
At a health center	188	39.5
Home and elsewhere	288	60.5
Total	476	100.0
		
Distance from village to health centre (range)		
0 (0 km)	181	38.0
1 (1 to 8.3 km)	121	25.4
2 (8.4 to 16.6 km)	111	23.3
3 (16.7 to 24.9 km)	50	10.5
4 (more than 25 km)	13	2.7
Total	476	100.0
		
Father's school attendance		
None	254	53.4
Muslim Koranic	71	14.9
Formal	151	31.7
Total	476	100.0
		
Mother's school attendance		
None	355	74.6
Muslim Koranic	41	8.6
Formal	80	16.8
Total	476	100.0
		
Mother's attendance in literacy classes		
No	424	89.3
Yes	51	10.7
Total	475	100.0
		
Father's religion		
Muslims	295	62.0
Christians	140	29.4
Animist and other religion	41	8.6
Total	476	100.0
		
Monogamous		
No	209	43.9
Yes	267	56.1
Total	476	100.0
		
Polygamous		
No	292	61.3
Yes	184	38.7
Total	476	100.0
		
Household ethnic group		
Bwaba	107	22.5
Marka	205	43.1
Mossi	94	19.7
Foulani	24	5.0
Samo	39	8.2
Others	7	1.5
Total	476	100.0
		
Father's profession		
Agriculture	412	86.6
Others	64	13.4
Total	476	100.0
		
Quartiles of revenue (In CFA francs)		
1st (0 to 146475)	119	25.0
2nd (146475.1 to 332125)	119	25.0
3rd (332125.1 to 595375)	119	25.0
4th (595375 to Highest)	119	25.0
Total	476	100.0

### Immunization coverage and knowledge about vaccination activities

The complete immunization coverage was 50.2% (CI: 45.71-54.69) with 2.52% (CI: 1.10-3.90%) having never been vaccinated. Table 2 reveals that 59.7% of parents (CI: 45.71-64.11) knew that the objective of vaccinating children was to prevent disease, while 10.29% (CI: 7.57-13.03) of parents said they had no idea about the objectives of vaccination; for others, all vaccinations are to prevent specific diseases like poliomyelitis. Vaccination records were available for 73.3% of children. The majority of respondents identified poliomyelitis (66.4%) as the primary example of vaccine-preventable disease, followed by malaria (31.87%). Tuberculosis (1.87%), whooping cough and diphtheria (<1%) were given less consideration as preventable diseases.

**Table 2:** Objectives of immunization according to respondents.

**Objectives of immunization**	**Numbers of respondents**	**Percentages (%)**	**95% CI**	
Don't know	49	10.29	7.57	13.03
Prevent disease	284	59.66	55.29	64.11
For health (without precision)	136	28.57	24.54	32.66
It's for a specific disease (mainly polio)	7	1.48	0.41	2.59
Total	476	100		

Respondents were asked about specific concerns that prevented them from participating in vaccination sessions. From the 476 respondents, 318 (66.8%) mentioned communication problems (they did not understand what the health workers wanted; they thought their child was totally immunized); 5% (25/476) complained about the organization of immunization sessions (e.g. "*health workers don't stay in the village for enough time; they come too late, they discriminate against some children").*

### Factors associated with complete immunization coverage

#### Knowledge, vaccination documents and immunization uptake

Knowledge about the reasons for immunization and complete immunization coverage were found to be associated with the parents' level of education. Children of non-educated fathers who reported no knowledge of the objectives of immunization were less likely to be completely vaccinated (Table 3). Although there was a strong relation between the availability of vaccination documents and complete vaccination status, this relation was only significant in rural areas and not significant for children of the highest income group (p < 0.001). In rural settings, the perception of communication problems between parents and health workers was significantly associated with complete immunization coverage. There was no significant association with those living in the urban area of Nouna.

**Table 3:** Parents' knowledge. Availability of booklet. Perception of communication problem and vaccination uptake.

	**Not completely vaccinated**	**Completely vaccinated**	***p *≤ 0.05**
**Knowledge of the preventive objectives of immunization**			
***Illiterate father***	**Total = 325; n (%)**		
Don't know or it's for a specific disease	27 (15.7%)	12 (7.8%)	
To prevent diseases or for child health	145 (84.3%)	141 (92.2%)	0.030
			
**Availability of a vaccination record document**	**Total = 357; n (%)**		
No	68 (35.6%)	29 (17.5%)	
Yes	123 (64.4%)	137 (82.5%)	0.000
			
**Perception of communication problems**			
***Rural area***	**Total = 349; n (%)**		
Did not perceive communication problem	41 (24.8%)	80 (43.5%)	
Perceived communication problem	124 (75.2%)	104 (56.5%)	0.000

#### Geographic factors, locality, birthplace, distance of vaccination site and immunization

Table 4 shows that children in rural areas have a more complete immunization coverage rate than those in the urban area among non-educated fathers and mothers (p = 0.028 for fathers and 0.026 for mothers). Children born at health facilities in the villages have a more complete immunization coverage rate compared to those born at health facilities in Nouna town (52.5% versus 47.5%, p = 0.003).

**Table 4:** Relation between locality, birth place, distance to the vaccination site and vaccination uptake.

**Locality**	**Not completely vaccinated**	**Completely vaccinated**	***p *≤ 0.05**
**Urban/rural**			
***Illiterate father***	**Total = 325; n (%)**		
Urban	48 (27.9%)	27 (17.6%)	
Rural	124 (72.1%)	126 (82.4%)	0.028
***Illiterate mother***	**Total = 396; n (%)**		
Urban	55 (26.7%)	33 (17.4%)	
Rural	151 (73.3%)	157 (82.6%)	0.026
			
**Birth place**	**Total = 476; n (%)**		
Born at health facilities	87 (36.7%)	101 (42.3%)	
Born out of health facilities	150 (63.3%	138 (57.7%)	0.215
			
***Born at health facilities***	**Total = 188; n (%)**		
Urban	60 (69.0%)	48 (47.5%)	
Rural	27(31.0%)	53 (52.5%)	0.003
			
***Born outside health facilities***	**Total = 288; n (%)**		
Urban	12 (8.0%)	7 (5.1%)	
Rural	138 (92.0%)	131 (94.9%)	0.317
			
**Distance from household to vaccination site**	**Total = 476; n (%)**		
1 0-250 meters	83 (35.0%)	89 (37.2%)	
2 >250-500	64 (27.0%)	72 (30.1%)	
3 >500-750	50 (21.1%)	41 (17.2%)	
4 >750-1000	19 (8.0%)	21 (8.8%)	
5 >1000 +	21 (8.9%)	16 (6.7%)	0.674
			
**Distance from village to health center (range)**			
***Rural***	**Total = 349; n (%)**		
0 (0 km)	22 (13.3%)	47 (25.5%)	
1 (1 to 8.3 km)	59 (35.8%)	47 (25.5%)	
2 (8.4 to 16.6 km)	49 (29.7%)	62 (33.8%)	
3 (16.7 to 24.9 km)	29 (17.6%)	21 (11.4%)	
4 (more than 25 km)	6 (3.6%)	7 (3.8%)	0.015

The mean distance from households to vaccination site was 453.7 m; 432.5 m (SD: 476.2) for completely vaccinated children and 475.16 m (SD: 400.23) for not completely vaccinated ones. As to intra-village variation, children in close proximity to the vaccination site had no advantage in terms of complete vaccination coverage rate.

Considering the distance between the village of residence of the child and the health centre, the mean distance was 6.8 km (SD: 7.67); 6.6 (SD: 7.69) for vaccinated and 7.0 (SD: 7.66) for not completely vaccinated. There was a significant difference between the distance from the child's village to the health centre and immunization uptake (χ^2 ^= 12.298, df = 4; p = 0.015). The correlation between the range of distance from village to health centre (dichotomized in 0 = 0 and 1 to 4 = 1) and complete immunization coverage is significant (r = -0.153, at p = 0.01 level; Table 5). Rural children living in villages hosting the health centres tend to have better coverage.

**Table 5:** Correlations between study variables (rural areas).

	**1**	**2**	**3**	**4**	**5**	**6**	**7**	**8**	**9**	**10**	**11**	**12**	**13**
1 Complete vaccination	1												
2 Knowledge of the objectives of vaccination	0.072	1											
3 Perception of communication problems	-0.195**	-0.017	1										
4 Availability of vaccination record card	0.216**	-0.043	-0.127*	1									
5 ATRA (8.3 km)	-0.153**	-0.045	0.001	-0.179**	1								
6 Birth place	-0.148**	-0.031	-0.025	-0.147**	0.756**	1							
7 Father's religion	0.163**	0.159**	-0.029	-0.055	-0.308**	-0.242**	1						
8 Education of the father	0.074	-0.018	-0.169**	0.136*	-0.198**	-0.110*	0.201**	1					
9 Education of the mother	0.096	0.063	-0.146**	0.092	-0.154**	-0.140**	0.178**	0.224**	1				
10 Adult literacy of the mother	0.115*	0.049	0.089	0.067	-0.142**	-0.154**	0.208**	0.037	0.139**	1			
11 Monogamous marital status	0.148**	0.052	-0.110*	0.041	-0.050	0.008	0.193**	0.052	-0.006	0.084	1		
12 Polygamous marital status	-0.133*	-0.042	0.084	-0.050	0.042	-0.037	-0.213**	-0.104	-0.010	-0.088	-0.900**	1	
13 Economic status	0.133*	0.012	0.017	0.041	-0.049	-0.046	-0.028	0.082	0.094	0.001	-0.119*	0.147**	1

** The correlation is significant at 0.01 level (bilateral).

The correlation is significant at 0.05 level (bilateral).

Variable ATRA dichotomised in 0=0 and ATRA 1 to 4=1.

#### Social factors (education, religion, marital status) and immunization uptake

Table 6 shows children from non-educated fathers were less immunized in the urban area (n = 127). In rural settings, the adult literacy of the mother was found to significantly determine vaccine uptake; 13.7% of the children of literate mothers were completely vaccinated compared to 6.7% of the non-vaccinated (p = 0.032).

**Table 6:** Relation between social factors and vaccination uptake.

**Variables**	**Not completely vaccinated**	**Completely vaccinated**	***p *≤ 0.05**
***Urban***			
**Education of the father**	**Total = 127; n (%)**		
Not educated	48 (66.7%)	27 (49.1%)	
Educated	24 (33.3%)	28 (50.9%)	0.046
**Education of the mother**	**Total = 127; n (%)**		
Not educated	55 (76.4%)	33 (60.0%)	
Educated	17 (23.6%)	22 (40.0%)	0.047
			
***Rural area***			
**Mother's attendance in literacy classes**	**Total = 348; n (%)**		
Illiterate	154 (93.3%)	158 (86.3%)	
Literate	11 (6.7%)	25 (13.7%)	0.032
**Membership of the mother in associations**	**Total = 474; n (%)**		
Not member	189 (80.1%)	178 (74.8%)	
Member	47 (19.9%)	60 (25.2%)	0.168
			
***Rural area***			
**Monogamous parents**	**Total = 349; n (%)**		
No	88 (53.3%)	71 (38.6%)	
Yes	77 (46.7%)	113 (61.4%)	0.006
**Polygamous parents**	**Total = 349; n (%)**		
No	87 (52.7%)	121 (65.8%)	
Yes	78 (47.3%)	63 (34.2%)	0.013
**Religion **(rural three lowest economic quartiles)	**Total = 253; n (%)**		
Muslim	85 (65.4%)	60 (48.8%)	
Others	45 (34.6%)	63 (51.2%)	0.011

After controlling for both locality (rural/urban), and economic status, we notice that in rural areas, in the poorer three quartiles, children from Muslim families had lower immunization coverage rates (48.86%) compared to others (51.2%) (p = 0.011 n = 253). Children of polygamous fathers were more likely to have an incomplete vaccination status in rural areas. Marital status and religion were not significantly related to lower immunization coverage rate in the urban area.

#### Economic factors and immunization uptake

With regards to the principal economic activities of parents, no significant difference was noticed between children of farmers and others. Table 7 provides the analysis of the core revenue of the household. After controlling for locality and education, it appears that children of non-educated fathers among the higher fourth quartile (households earning more than 595375 CFA francs/year) had better immunization coverage compared to children of non-educated fathers among the poorer three quartiles (earning less than 595375 CFA francs/year). Children of non-educated fathers of the forth quartile represent 32.5% of the completely vaccinated group and only 17.7% of the unvaccinated group (p = 0.017) in rural settings (n = 250).

**Table 7:** Relation between economic status and vaccination uptake.

**Variables**	**Not completely vaccinated**	**Completely vaccinated**	***p *≤ 0.05**
**Profession *All participants***	**Total = 476; n (%)**		
Farmers	209 (88.2%)	203 (84.9%)	
Others	28 (11.8%)	36 (15.1%)	0.299
			
**Economic status of household**			
*Rural area, non-educated*	**Total = 250; n (%)**		
- 1^st ^to 3^rd ^quartile (< 595375 CFA/year)	102 (82.3%)	85 (67.5%)	
- 4^th ^quartile (> 595375 CFA/year)	22 (17.7%)	41 (32.5%)	0.007

#### Characteristics of completely vaccinated children (focus on rural area)

This part of the analysis was restricted to rural areas (349 children). We excluded from this analysis those variables that were not associated with vaccination status in the preceding steps, such as sex, distance from the household to the vaccination site, membership of the mother with an association (societies), and principal activities of the father. The remaining variables were dichotomized. The Pearson correlation test was then performed with variables that showed to be related to the dependant variables. As presented in Table 5, from 12 independent variables, three variables (knowledge of the objectives of immunization, r = 0.072; education of the father, r = 0.074; and education of the mother, r = 0.097) showed no significant correlation with the immunization status. All these variables were excluded from further analyses. In addition, the mother's attendance in literacy classes was also excluded because of the small number of cases.

Before proceeding to our analysis, we split the remaining variables into two groups. Variables related exclusively to the household formed one group (religion, monogamous marital status, polygamous marital status, and economic status) and variables related to the health system formed the other group (perception of problem of communication, availability of vaccination document, distance to the health centre, and place of birth). A stepwise logistic regression was performed on vaccination status entering group 1 variables at the first step and group 2 variables at the second step.

The Hosmer-Lemeshow test of goodness of fit was not significant (khi2 = 5.516, df = 7; significance = 0.597), indicating that the model fits the data. The Nagelkerke R2 is 0.186; the total percentage of correct classification of the model is 66.2% (the intercept only model was 52.7% and the step 1 model indicated 61% of correct classification). Results from the final model, in Table 8, suggest that children from households where vaccination documents were available are 2.4 times more likely to be in completely vaccinated groups (OR = 2.381; 95% CI = 1.436-3.948). The perception of communication problems by parents decreases the chance of being completely vaccinated by 0.46 (OR = 0.461; 95% CI = 0.283-0.750) and the household being in the forth quartile of the economic strata increased the likelihood of complete vaccination by 2.1 (OR = 2.1; 95% CI = 1.24-3.55). Being of non-Muslim religion increased the chance of being in the completely vaccinated group by 1.8 (OR = 1.811; 95% CI = 1.102-2.985).

**Table 8:** Logistic regression model: vaccination status and predicting factors.

**Variables**	**B**	**Significance level**	**O.R (CI for O.R 95.0%)**
Father's religion (non Muslim)	0.595	0.019	1.813 (1.102-2.985)
Economic status (4th quartile)	0.742	0.006	2.100 (1.242-3.554)
Availability of vaccination record card (Yes)	0.868	0.001	2.381 (1.436-3.948)
Perception of communication problems (Yes)	-0.775	0.002	0.461 (0.283-0.750)

## Discussion

The complete immunization coverage rate (50.2%) remains low in Nouna district with many children reaching their first birthday without any contact with immunization services. Our findings, however, show a significant improvement from the preceding year's rate of 31.5%, obtained from the census of the district [22]. These findings require recognition of the limitations of the study and determination of the relevance of the results.

### Knowledge, vaccination documents and importance of communication in immunization uptake

The relevance of the findings could be reduced if the relationship between immunization and the availability of vaccination record documents was related to our data collection procedures. Thus this finding requires further discussion. The relationship between immunization and the availability of vaccination record documents suggests three interpretations. The first is recall bias; the eligible age group for the study included children who had already left the immunization program. Accurate recall by parents of events that took place almost a year before may, in some cases, be compromised. Challenging this hypothesis, however, is the fact that our analysis shows that the association was only significant in low economic groups and in rural areas. Additionally, of the 26.7% of those with no vaccination document, 2.5% claimed their child got no vaccine while 17.0% had no documentation, but were, in fact, completely vaccinated following appropriate mothers reporting [46,47]. The influence of not having a document on reporting, therefore, appears to be negligible.

A second interpretation, and important consideration, is that the children of parents who lost or could not afford immunization documents are not accepted at vaccination sessions. In many rural areas health workers do not vaccinate children who do not have vaccination cards. Some analyses of the anthropological study that was also carried out by CRSN as part of this operational research grant [28] show that mothers who lose their vaccination booklets or bring damaged documents to immunization sessions feel humiliated by health personnel. Vaccination workers complain they cannot interrupt the vaccination session in order to help mothers recall their children's vaccination history.

Our third interpretation is that economic conditions affect the ability to afford and keep immunization record documents in good condition.

More than half of the respondents had an accurate idea about the objectives of vaccination; about 60% of the respondents know that immunization is to prevent disease. Referring to the lower limit of the confidence interval (55.3-64.1) of this estimate, however, we suggest the urgent need for better information for at least 45% of the population. While people recognize that vaccination is for the health of their children, more detailed knowledge about immunization might be required. UNICEF, for example, states that "It is essential that all parents know why, when, where and how many times the child should be immunized. Parents also need to know that it is safe to immunize their child even if the child has an illness or a disability or is suffering from malnutrition" [20]. Reinforcing knowledge about the goal of immunization is crucial, exemplified in our findings by a significantly higher rate of complete immunization coverage when non-educated parents understand the preventive goals of vaccination (p = 0.03; Table 3).

Knowledge about child preventable diseases reflects an understanding of the immunization goals. Apart from poliomyelitis, which is understood by more than half of respondents, other preventable diseases remain largely ignored. Knowledge about the immunization program is proportionate to the effort the health system deploys for communication and promotion of awareness. Diseases that are addressed by specific awareness and campaign programs, such as is done for poliomyelitis, are better known because of their extensive coverage in the media.

The vast amount of respondents who wrongly named malaria as an EPI preventable disease is also important. As reported elsewhere [33], it seemed unacceptable for a frequent and disabling disease like malaria not to be taken into account by EPI. Many other diseases or symptoms considered as preventable, such as cholera or headaches, are not within the mandate of EPI. If participation in immunization is dependent on the expectation that all diseases and symptoms will be controlled, the trust and confidence of those uninformed participants who suffer - after being inoculated - from diseases with similar symptoms that were not part of the vaccination regimen will be lost. The EPI in Burkina Faso has been extended to Hepatitis B, and meningitis from *hemophilius influenzae *following our study period. Extending EPI to these new diseases offers considerable improvement that may also increase the populations' participation in the program if they are well informed.

Our results show that perception of communication problems by parents halves the chance (0.46 times) of a child being completely vaccinated. We suggest, along with others, that better communication, including more appropriate interaction between parents and health workers is needed [9,31]. Communication on immunization in the Nouna district is rarely comprehensive; it is generally marginal, partial and sporadic. Campaigns on specific diseases like poliomyelitis and meningitis overshadow the whole EPI. In addition, health workers insist they are not able to engage in communication/health education during immunization sessions; they are overloaded with the responsibilities of registering children, filing records, managing and administrating vaccines. New strategies are needed to make communication an integral part (not a marginal component) of the immunization program in order to achieve the target proposed by UNICEF [20]. This may require consideration from decision makers regarding the actual human resources and service needs of the health centres, which might be the first step towards the essential recognition of immunization as a public health priority in Burkina Faso.

### Importance of education literacy and religion

Our analysis revealed the considerable influence of social factors contributing to vaccination status. Parents who attended school (in the urban area) and mother's attendance in literacy classes (in rural areas) were related to vaccination status. The influence of education confirms findings from previous studies [18]. Little is known, however, about the relationship between parents' attendance in literacy classes and immunization status of the child. This suggests the need to assess the relationship that might be built between immunization communication strategies and current strategies used in literacy training when designing immunization coverage improvement interventions. Increasing the level of adult literacy or incorporating vaccination awareness in literacy programs may improve the understanding of rural communities on health issues such as immunization.

Another social determinant assessed in this study was religious affiliation. Particular Muslim factions shaped some communities' relation to immunization questions [14,15]. In our study, children of Muslim families (controlling for economic status) have significantly lower rates of complete immunization coverage in rural areas. Non-Muslims had almost twice the probability of being in the completely vaccinated group. Our study did not account for the role of Muslim opposition to immunization that played out in Nigeria (where immunization was presented by some Islamic factions as an instrument threatening the well-being of Muslim communities) [14,15]. We suggest, however, that in Nouna the problem is more related to access to information. A previous study in the area reported that women who attended Koranic School were less likely to participate in HIV counselling [45]. In Nouna district, women are responsible for going with the child to the vaccinating site. In some Muslim communities, external informants have only limited and controlled access to women. In addition, in the two Muslim dominant ethnic groups (Marka and Mossi), women are said to be less "free" than in the Christian and the animist dominant ethnic group (Bwaba) [44]. Our result corroborates previous findings as to the sensitivity of the relationship between immunization uptake and religious matters [12,17]. The problem is not limited to Muslims, as researchers [17] have also noted the low immunization coverage rates among orthodox Protestant inhabitants in the Netherlands. In Nouna district, we have previously documented the influence of the Catholic church on AIDS prevention campaigns, particularly its untactful disapprobation of condom use [44]. Combining these findings, it appears that the complex relationships between religious matters and health outcomes must be questioned more deeply. These results suggest that intervention on the issue can neither neglect religious considerations nor the particular learning environments of specific groups. Health intervention planners should integrate both health promotion and adult literacy into their activities; they must also consider the distribution and involvement of religious groups.

### Distance and location

Our study put an emphasis on location and geographic determinants, and we can draw three conclusions from these factors. First, unlike other findings [23-25], there is no intra-village distance-based disparity as to children's vaccination status. Those living at village boundaries have the same probability of being fully vaccinated as those living near the selected fixed vaccination sites of the village. The average distance separating the households of completely vaccinated children and those separating others from the vaccination points are not statistically significant. However, the result is strategically significant as it argues in favour of the current vaccination strategy in Nouna district. In each village, one, two or three vaccination sites are selected with the participation of the community; these places change according to the season. We can also postulate from this result that the withdrawal of some of these sites, which is planned by some health teams, may influence vaccination coverage in those areas. The second conclusion related to location and geographic factors is that after controlling for urban areas, our analysis suggests that children of the villages hosting a health centre have better immunization coverage rates compared to surrounding villages, but there is no difference between villages outside the site of the health centres. Equal effort is given to all outreach villages. The third conclusion suggests that children born in health facilities in the villages have a better vaccination coverage rate compared to those born at health facilities in the town of Nouna. This may show less effective targeting of services in the larger, more heterogeneous communities. Unlike many cases where urban areas are better off with respect to immunization coverage, living in Nouna does not warrant better immunization coverage compared to rural areas. Discussions with some health workers suggest that in the urban area of Nouna, some nurses regard immunization as a low status activity. There is a need for district managers to design specific interventions for towns in similar conditions so that the view that immunization is an important health intervention can be restored at the health worker level.

### Economy and living conditions

Like previous studies [19], our findings suggest there is a difference in vaccination coverage related to the economic conditions of households. In rural areas, children in the highest economic quartile have a better immunization coverage rate and a greater probability (2.1 times) of being vaccinated. However, we should not reduce the ability to pay to an incentive to immunize. The influence of economic factors remains more complex than ability to pay, as immunization services are free of charge in Burkina Faso. At the same time, it is also difficult to claim that all health centres are following this free of charge requirement. Some of the reasons given by mothers for not participating in immunization sessions are that they did not have the money required (suggesting their belief that money is sometimes being demanded of them). The indirect influence of economic factors on immunization at household levels is a more obvious explanation. When the household is experiencing food and resource shortages, participating in a session becomes a matter of lesser priority. A man who participated in our discussion sessions gave a clear explanation:

*"What I add...it's the problem we usually face during rainy season. In the household we often face difficulties, i e. some crisis periods, when there is no food to eat. When we spend a bad night because we had no more supplies, each may try *(in the morning) *to find something for the children. So you are all in a hurry; the husband will go on his way and the wife will try to find some shea nuts *(in the bush). *Under the pressure of food shortage, as parents, you don't want children to wakeup and find you without a solution for their hunger; they will look so pitiful. These problems can be the reason for not respecting the appointment with the vaccination team." *A young father in Toni village.

It may be difficult for decision makers to control the indirect influence of economic factors on immunization uptake. However, there remains a need to identify all the interactions between the health system and the communities that require money. Thus, large-scale communication about the free services and careful monitoring of vaccination procedures should be undertaken to clarify the issue at the community level.

### Final considerations

The result of the regression model reported a Nagelkerke R2 of 0.186; although this may explain only 18.6% of the variance of immunization status in rural areas, we suggest this is an important contribution. Given the equal distribution of vaccination outcome (50.2%), the variance is at its maximum and explaining 18% of that variance is critical. In addition, a child's immunization uptake depends on many other factors not related to communities; this also needs to be taken into account when explaining the overall variance of complete immunization outcome. Finally, the overall validity of the regression is proven by a non-significant Hosmer-Lemeshow test of goodness of fit (khi2 = 5.516, df = 7; significance = 0.597) [48,49].

Although research from the health services perspective would have suggested a different explanation of the variance, we can still conclude from this discussion that the result of the study is relevant and can orient intervention.

The results of the regression model distinguished two groups of factors influencing immunization coverage. Two factors are related to households (economic conditions and religion) and two other factors related to the interaction between households and the health centre (communication and availability of vaccination record document). Our research explored the question from a population perspective. Our results suggest that considering both communities and health services is important in designing interventions. An intervention targeting only the community or only the health workers will not resolve the low immunization coverage rate. There is a need for an integrated approach at both the community and the health service level.

Based on our results and analysis, we can postulate that the intervention planned by CRSN and the Nouna health district should have at least two principal components. One should target the community and the other one should be at the level of the health service delivery. Health workers must be trained to fully integrate communication into their activities and appropriate communication frameworks should be established between health workers and communities. Designing and adapting culturally appropriate sensitization tools that incorporate the use of pictures would probably address issues related to illiteracy. It is important that information about the immunization program be as complete as possible, that information be made available in all public places and that it be relevant to all residents. Collaboration with religious and community leaders is also essential to ensure broad dissemination of immunization messages. To reinforce the importance of immunization at both community and health workers levels, trainings planned as part of the intervention are expected to inform all participants about the real cost incurred by the government and its partners for immunization. Community members, however, should be well informed that, despite this cost incurred by the government, immunization is free of charge; this will allow them to recognize the effort made to bring immunization to their doors while at the same time making them more cautious of attempts to make them pay to immunize their children.

### Study limitations

A key issue faced by immunization researchers in areas with high illiteracy rates is managing respondents' recall bias, and information bias when using only vaccination cards. Studies show that mothers' responses are accurate and provide generally adequate information even if they are said to underreport immunization uptakes [46,47]. The rate for immunization coverage we obtained in this study is extremely high compared to results of the national census of the previous year. Concern about the under reportage of immunization coverage due to poor recall has been put to rest. What this study cannot rule out is the possibility of over reportage due to poor recall. We show, however, a strong association between complete immunization and the presence of immunization booklets. This study cannot, on its own, provide an explanation for the increase in complete immunization coverage rate nor can it completely account for the role of the vaccination card in these results. The anthropological study mentioned earlier [28] and which had a special focus on the immunization record cards provides a fuller description to that end.

It was not our original intention to compare urban and rural areas and therefore the sampling procedure did not take this into account. Determining the number of children proportionally resulted in having a relatively fewer number of children in the urban area. The result is that the regression model could not accommodate locality (urban/rural) for categories and use at the same time all the eight variables having a significant relationship with the outcome. Two variables distribution in the urban area could not satisfy the rule of minimum ratio of 10 to 1 (having a sample size with at least 10 cases for 1 variable in the regression) [49]. As such, extending the interpretation of the regression analysis to the urban area requires caution. It is important to note, however, that the analysis of six variables in the regression (not presented here) produced the same result as the one presented in this report.

Another study limitation is the procedure used to determine economic status. Determining economic status through a single monetary value estimated from agricultural production, animals and poultry, salaries and trade revenues is rather exclusive. Equipment, housing conditions and others properties (also collected during the study) could have contributed to the reliability and validity of socio-economic status. Additionally, the principal economic activity was the main source of information, excluding the secondary activities of other members of the household. Nonetheless, our estimate of economic conditions is associated with education and knowledge variables, providing external validity.

## Conclusion

Beyond reaching the communities, the primary goal of EPI activities should be to get people to better understand what vaccination is about and what is at stake. Poor communication around immunization and inadequate knowledge about its objectives and the importance of the immunization booklet seem to account for the low immunization coverage in our study area. Comprehensive information and communication on immunization (instead of relying on sporadic single disease specific messages like those of poliomyelitis and other epidemics) may improve understanding of immunization for many communities; both strategies must be used complementarily. The question of whether this communication work is feasible for small health teams needs to be addressed.

Social factors like education are always important with regards to access and health seeking behaviours, including immunization uptake. In the context of a high level of illiteracy, as occurs in the Nouna Health District, taking note of adult literacy and accommodating it through health promotion mechanisms would be an appropriate approach to improving the immunization coverage rate. A clear difference among certain religious groups was found in immunization coverage. We suspect that unequal access to information is the likely cause of this difference. Designing local interventions should therefore take into account complex cultural specificities to access, such as religion.

The goal of improved access is currently also hampered by poor household economic conditions. Health intervention planners have limited influence on economic conditions of the households. However, considering critical economic periods and conditions in the implementation of interventions may help solve this limitation.

As to geographic factors, the incorporation of vaccination strategies using local vaccination sites to target uptake in remote areas of the district provides better opportunity to access vaccination services for children. Consequently, rural areas are in a better position to achieve improved immunization coverage. Different approaches are clearly needed for the urban area. Urban health units need to make additional efforts to better address the needs of a more heterogeneous range of people living in urban centres, starting with children born in their health facilities.

Our findings suggest that improving immunization coverage requires considering contextual factors related to individual resources and communities but also those related to the interaction between communities and the health system. Determining the responsibility and capability of each partner is a key to designing contextually relevant health interventions.

## Ethical issues

This research was accepted by the local ethical committee (Comité Local d'Éthique). Interviews were performed after explaining the objective of the study and obtaining the consent of the respondents. Security and confidentiality of the data is preserved using the CRSN procedures. The informants are rendered anonymous by using an alphanumerical coding system to identify the compound, the household and the members.

## List of abbreviations used

WHO: World Health Organization; EPI: Expanded Program on Immunization; IDRC: International Development Research Centre; CIII2: Canadian International Immunization Initiative Phase 2; CRSN: Centre de Recherche en Santé de Nouna; CMA: Centre de Santé avec Antenne Chirurgicale; CSPS: Centre de Santé et Promotion Sociale; DSS: Demographic Surveillance System; GPS: Global Positioning System; ATRA: Average Theoretical Range of Action.

## Competing interests

The authors declare that they have no competing interests.

## Authors' contributions

AS participated in the design of the study, carried out the field studies, analyzed the data and drafted the manuscript. SS carried out the GPS and the field measurements of distances for the study. BK participated in the coordination of the study, the critical revision and the interpretations of the results. MD participated to the critical revision and interpretation of findings. JG participated to critical revision, interpretation of the findings. GB conceived of the study, and participated in its design and coordination and helped to draft the manuscript. All authors read and approved the final manuscript.

## Supplementary Material

Additional file 1 Abstract in French. Click here for file
