# Supplementary material for: Assessment of factors associated with complete immunization coverage in children aged 12-23 months: a cross-sectional study in Nouna district, Burkina Faso
Source: BMC Int Health Hum Rights. 2009 Oct 14;9(Suppl 1):S10. doi: 10.1186/1472-698X-9-S1-S10 (PMC2762310; doi:10.1186/1472-698X-9-S1-S10)
Supplement: Additional file 1 — Abstract in French. [file 1472-698X-9-S1-S10-S1.pdf]

Analyse des facteurs associés à la couverture vaccinale complète chez les enfants âgés de 12 à 23 moi: étude transversale à Nouna, Burkina Faso.

Aboubakary Sanou, Seraphin Simboro, Bocar Kouyaté, Marylène Dugas, Janice Graham, Gilles Bibeau.

Résumé

Problématique

Le Programme Élargi de Vaccination (PEV) reste encore perfectible. Dans des pays comme le Burkina Faso, la couverture vaccinale dans le district sanitaire de Nouna était en 2003 de 31,5% contre 52% au niveau national. Cette étude identifie les facteurs spécifiques liés au niveau de la couverture vaccinale dans le district sanitaire de Nouna en vue de proposer une stratégie d'amélioration de cette couverture dans ce district et dans d'autres districts aux conditions environnementales et sociales similaires.

Méthodes

Une étude transversale a été menée dans 41 communautés rurales et une zone semi urbaine. Les données se rapportent à 476 enfants âgés de 12 à 23 mois issus d'un échantillon représentatif de 489 enfants tirés de la base de données du Système de Surveillance Démographique (SSD) du Centre de Recherche en Santé de Nouna (CRSN). Une analyse du parcours de vaccination de ces enfants a été réalisée. Les relations entre le statut vaccinal et les variables socio-économiques et diverses variables contextuelles liées à leurs parents et aux ménages ont été analysées à l'aide des statistiques du Chi-carré, de la corrélation de Pearson et de la régression logistique.

Résultats

Le niveau de couverture vaccinale complète était de 50,2% (IC, 45.71; 54.69). La connaissance des parents de la valeur préventive de l'immunisation était positivement associée au statut de couverture vaccinale complète ($p = 0.03$) en milieu rural. Les enfants de parents ayant évoqué une perception d'un problème de communication autour de la vaccination avaient un plus faible taux de couverture vaccinale ($p < 0.001$). Il n'y a pas de différence due à la distance à l'intérieur des villages eux-mêmes et entre les villages hors du site de la formation sanitaire. Les enfants des parents non scolarisés ont un meilleur taux de couverture vaccinale en zone rurale qu'en zone urbaine ($p. = 0.028$). Une bonne communication autour de l'immunisation et de l'importance de la carte de vaccination, ainsi que les facteurs économiques et religieux influencent le statut vaccinal des enfants.

Conclusions

Les sites de vaccination dans les zones rurales ont pour but de donner aux enfants une plus grande opportunité d'accès aux services de vaccination. Cependant, ces efforts sont souvent entravés par les mauvaises conditions économiques, le faible niveau des connaissances et de communication sur la vaccination. Une communication globale sur la vaccination pourrait améliorer les connaissances des populations sur le sujet. Il est tout de même important que les interventions locales prennent en compte les particularités religieuses et les moments de l'année présentant des difficultés économiques. Des approches spécifiques qui prennent en considération ces distinctions doivent être appliquées dans le milieu rural et en milieu urbain.
